# Effect of friction stir processing parameters on mechanical properties of different aluminum alloy plates

**DOI:** 10.1038/s41598-025-11948-1

**Published:** 2025-07-25

**Authors:** Mohamed S. El-Sayed, Masoud Ibrahim, Ahmed I. Abdel-Aziz

**Affiliations:** Mechanical Engineering Department, Faculty of Engineering, Fayoum University, Fayoum, Egypt

**Keywords:** Aluminum alloys, Friction stir process, Response surface methodology, Mechanical properties, Engineering, Materials science

## Abstract

This research presents a pioneering investigation into friction stir processing (FSP) of different aluminum alloys reinforced with silicon nitride powders (Si_3_N_4_). By optimizing FSP parameters, such as stir tool profile and cooling medium, this study aims to enhance the microstructure and mechanical properties. The findings of this research contribute to the development of advanced FSP techniques for improving the performance of these alloys in different industries. FSP of three aluminum alloys (1050, 2011, and 6063) reinforced with Si_3_N_4_ powders was conducted under different processing parameters was investigated. Tensile tests and hardness evaluations were conducted to assess the mechanical properties. The best combinations of processing parameters were defined using the Taguchi L^27^ orthogonal array, while a response surface methodology (RSM) with a central composite design of three factors and three levels was employed to develop the relationship between the FSP parameters (material type, pin profile, and cooling rate). The selected outputs included yield strength (YS), ultimate tensile strength (UTS), hardness (Hv), and elongation percentage (El%). An analysis of variance (ANOVA) was conducted to identify the significant process parameters affecting the responses. The results after FSP on Al plates indicate that the optimum UTS and YS values are achieved by performing FSP on 2011 AA with a conical pin profile and under indirect air-cooling. The highest hardness value was obtained under the same conditions but with indirect R-410 A cooling. Conversely, the optimum value of El% was reached by conducting FSP on 1050 AA with a cylindrical pin profile and indirect R-410a cooling. The maximum UTS, YS, El, and HV values are 286 MPa, 167 MPa, 40%, and 118 HV, respectively. Material type (M) was the primary dominant parameter affecting mechanical properties, while the cooling media ranked second.

## Introduction

Lightweight materials are the core of the aircraft and automobile industry. To investigate the worldwide defined concerns of hazardous, eliminating emissions, and irregular climatic fluctuations, the lightweight industry is important^1^. To achieve this goal, numerous searches and experiments have been carried out on topics of innovative and cost-effective manufacturing approaches for lighter structure materials. Aluminum (Al) and its alloys’ role in various engineering fields has become bigger due to their variable exceptional properties, like a great strength-to-mass ratio, good electrical conductivity, and adequate ductility^2–4^. Important industries like transportation, aircraft, and automotive consider Al and its alloys as the first choice for producing their different components and products^5–9^.

Aluminum matrix composites (AMCs) have gained significant attention in various industrial sectors due to their various combinations of strength, lightweight characteristics, and corrosion resistance. The addition of different reinforcement powders to Al alloy surfaces improves properties, such as increased hardness, tensile strength, and, sometimes, corrosion resistance^10–16^.

Friction stir processing (FSP) is a novel technique that can be used for the production of AMCs with a refined microstructure and adequate distribution of particles used as reinforcement^17–19^ FSP is a solid-state process that can achieve surface modification by causing high plastic deformation of the parent material and converting its structure into an ultra-fine grain structure. The degree of refinement depends on the type of additional powders (metal or ceramic) added to the metal matrix, and modification occurs only on the metal surface because the surface is only exposed to loads^20–22^ FSP uses a stir tool to mix the particles with the parent metal and convert coarse grains into ultra-fine grains^23^. The friction between the rotating tool and the metal surface causes heat, which softens the material (this temperature is below the melting temperature of the parent metal), and with the mechanical stirring produced by the tool, the material within the processed zone undergoes great plastic deformation, creating a dynamically recrystallized grain structure^24^. Large heat of FSP would cause a decrease in the strength of the heat-affected zone or thermo-mechanical affected zone, where the fracture often happens. This fracture occurs due to low dislocation density, precipitate phase dissolution, and grain coarsening. A high cooling rate during FSP could reduce the large heat effect^25,26^.

XuWF et al.^27^ investigated the effect of different cooling media (air and water) on the microstructural and mechanical properties of the friction stir welded (FSW) joint of 2029 AA. It was reported that water cooling enhanced the strength and ductility of the FSW joint because of the formation of fine grains, high-density dislocations, and a narrow or lesser precipitate deterioration zone by water cooling.

**Fig. 1:**
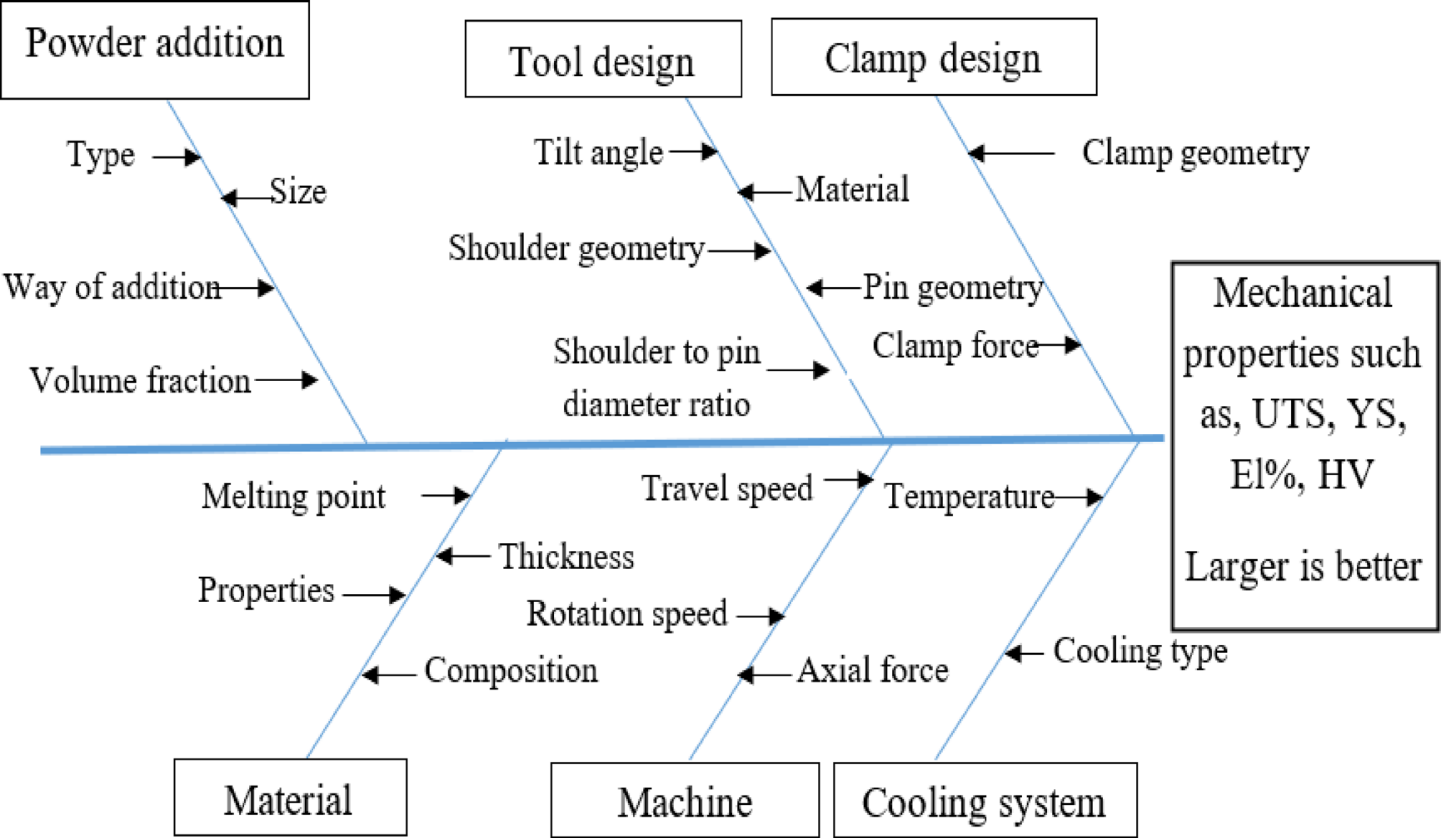
FSP variable parameters^36^.

The addition of reinforcement powder to the parent alloy could be done by filling a longitudinal groove parallel to the tool direction during processing or by creating blind holes in the parent metal surface. As mentioned in several previous studies, the variable parameters of friction stir processing (FSP) can be categorized into three classes: machine parameters, stir tool parameters, and cooling parameters. Notably, these studies have not investigated the effect of reinforcement type on the processed alloys. The FSP parameters are illustrated in Fig. 1. Any change of one parameter can produce a different property of the processed material^28^. Due to the complexity of FSP parameters, the optimization process of these parameters is very difficult. The development of an appropriate model that can predict process responses and their characteristics is very complex and differs from one way to another. Computational and analytical methods, or both experimental and numerical methods, were used for optimizing FSP parameters like the Taguchi approach^29–34^.

Response surface methodology (RSM) could be defined as a combination of mathematical and statistical techniques that drive the relationships between several hermeneutics’ variables and one or more response variables^35^. It is a sequential procedure that is used to clarify the relationships between several independent variables and dependent responses to optimize the response variable. It is used to detect the optimum conditions for a process or a system to ensure the best responses or results^36–40^.

Despite the large number of researchers who care about advances in the FSP method, the study of FSP parameters on various mechanical and microstructural properties is still in need of further surveys. Zeinelabdeen et al.^41^ studied the effect of FSP on the 6061 AA surface. In their research, a stir tool with a different rotational rate and fixed traveling speed was used. Corrosion resistance and microhardness of 6061AA alloy were evaluated as a result of the modification in the surface microstructure.

Ali Alavi Nia et al.^42^. searched the effects of pin geometry, advancing speed, and D/d ratio on the mechanical and microstructural properties of 6061 AA alloy under the FSP. Cylindrical, frustum, and prisms with triangular section, square, and hexagonal cross-section tool profiles were used. Three different traveling speeds and rotational rates were tested. The tensile strength and hardness of 6061 AA alloy were evaluated.

Ananda Kumar et al.^43^ used Central composite design (CCD) based on the response surface method (RSM) to study the effect of the addition of ceramic boron carbide (B_4_C) and Al oxide (Al_2_O_3_) particles to aluminum alloy. The experiments were done, and the effective process parameters were clarified as tool rotational rate, tool traveling speed, axial force, and reinforcement ratio. Genetic algorithms with RSM regression equations were used as a target function to identify ideal process parameters for the replies.

Tarunika Sharma et al.^44^. investigated the effect of boron nitride addition on the microstructure and mechanical properties of the 2024 AA surface. An FSP with a fixed travel speed and rotational rate tool was used to manufacture specimens. Tensile strength, hardness, and fatigue strength were measured to prove the enhancement in properties. Ramesh Arwin et al.^28^ used the FSP to incorporate silicon carbides (SIC) with 7050 AA. In their work, the effect of SIC weight (0 to 1%) on the microstructure and mechanical properties was indicated. Impact strength and hardness were affected by the addition of SIC particles.

Sumit Jain et al.^45^. conducted friction stir welding (FSW) to join 7075 AA alloy with 6061 AA with the addition of Al_2_O_3_ particles. In their work, the process parameters were rotational speed, traverse speed, and volume fraction of Al_2_O_3_ particles. A quadratic model was created to predict the responses of microhardness, tensile strength, and elongation% with a confidence level of 95%. Based on the previous review, there is a notable lack of studies investigating the effect of friction stir processing (FSP) parameters on the 2011 AA alloy. While some studies have explored FSP parameters on the 1050 AA alloy, the primary objective of this work is to examine the effect of FSP parameters on the microstructure and properties of (2011 AA, 1050 AA, 6063 AA) alloys, specifically in the context of aluminum matrix composites (AMC). Silicon nitride powders were added to these alloys during processing. The stir tool with different profiles (cylindrical, square, and tapered), and different cooling media (air, water, and R-410a) were considered as process variables. The microstructure and mechanical properties of these alloys were process outputs. Taguchi technique was used to define the optimum configurations, and RSM was used to predict the relationship between process variables and output responses (ultimate tensile strength (UTS), yield strength (Ys), elongation percent (El%), microhardness (HV) to gain optimal process parameters.

## Design of experiment

### Taguchi method

Taguchi handles quality in two main categories: offline and online stages during production^46^. The Taguchi method or technique depends on minimizing the variance of the response of interest, while the classical experimental design focuses on the average of the selected response. The ability of the Taguchi method to deal with single-response issues has enabled it to overcome much criticism for its several major limitations. The Taguchi optimization technique contains three main stages: system design, Parameter design, and tolerance design. The Taguchi optimization technique starts with identifying the quality characteristic and its noise factors or test conditions. Furthermore, the specification of the levels of each factor, and the experiment matrix are designed. Then, experiments and analysis of the gained data will be conducted. Finally, the performance of the levels is predicted^47^. The selection of an orthogonal array (OA) depends on the number of noise factors and their interactions, the number of levels for the selected factors, and the experiment’s cost limitations. As three levels and three factors are taken into consideration, L^27^ OA was used in this investigation. The factors and levels of each factor are listed in Table 1. The degree of freedom (DOF) for each factor is 2 (No. of levels − 1 = 2) and therefore the total degrees of freedom will be 6 (3* 2 = 6). In general, the DOF of the matrix must be higher than the total DOF of the selected factors. As the DOF of L^27^ is 26, it would be efficient for the investigation.

**Table 1:** Parameters and levels for experimental design.

Process parameters	Levels of factors		
	-1 / 1	0 / 2	1 /3
Material type	1050	2011	6063
Tool profile	Cylindrical	Conical	Square
Cooling rate	Air	Water	R-410a

### Mathematical modelling via response surface methodology (RSM)

Montgomery describes RSM as a set of mathematical and statistical techniques identifying the relationships between multiple explanatory variables and one or more response variables. This methodology is good for examining scenarios where several independent variables affect the dependent variables or responses, intending to optimize the response variable. RSM follows a sequential approach, which is used to identify the optimal operating conditions of a system or to define a range of factor space where the operating specifications meet requirements. In the current study, experimental investigations are structured using DOE method, employing a three-factor, three-level central composite circumscribed (CCC) design. To develop the mathematical model on this study, 20 experiments and three levels were used as shown in the design matrix of different runs as well as the levels of each parameter are demonstrated in Table 2. CCC designs offer high quality predictions over the entire design space and demands factor settings outside the range of the factors in the factorial design. The design consists of a full factorial 2^3^ = 8, plus 6 center points and six-star points (total 20 runs). +1 and − 1 were used as the lower and upper values of the level respectively.

**Table 2:** The design matrix using RSM.

Run No.	FSP parameters					
	M: Material type		*P*: Pin shape		C: Cooling type	
	Coded	Un codded	Coded	Un codded	Coded	Un codded
1	-1	1050	0	Conical	0	Water
2	0	2011	0	Conical	0	Water
3	0	2011	0	Conical	0	Water
4	0	2011	1	Square	0	Water
5	0	2011	0	Conical	0	Water
6	0	2011	0	Conical	0	Water
7	0	2011	0	Conical	0	Water
8	0	2011	0	Conical	-1	Air
9	1	6063	0	Conical	0	Water
10	0	2011	-1	Cylindrical	0	Water
11	0	2011	0	Conical	0	Water
12	1	6063	1	Square	-1	Air
13	-1	1050	1	Square	1	R-410a
14	1	6063	-1	Cylindrical	-1	Air
15	1	6063	-1	Cylindrical	1	R-410a
16	1	6063	1	Square	1	R-410a
17	-1	1050	-1	Cylindrical	-1	Air
18	-1	1050	1	Square	-1	Air
19	0	2011	0	Conical	1	R-410a
20	-1	1050	-1	Cylindrical	1	R-410a

The response surface of FSP was ultimate tensile strength (UTS), yield strength (YS), and elongation percentage (El%). The developed model was used to predict the response (outputs), which clarifies the dominant parameter (Material type (M), pin profile (P), or cooling rate (C) as shown in Table 2. Quadratic and two-way interaction effects of the input process parameters on the mechanical properties were estimated by regression analysis of the data obtained from experiments. The load used to run all 20 experiments on the milling machine was about 5 KN. FSP parameters and levels for experimental design using central composite design are listed in Table 1. Three different Al alloys were used (1050 AA, 2011 AA, 6063 AA), three different profiles of the tool were selected (cylindrical, tapered, and square), and cooling media with varying capacities of cooling were selected (air, water, and R-410a).

Representing the response Y (UTS, YS, and El%), the response is a function of material type (M), pin profile (P), and cooling rate (C), and it can be expressed as Y = f (M, P, C).

The second-order polynomial (regression) equation used to represent the response surface is given by^48^

1 $$\:Y={b}_{0}+\sum\:\left({b}_{i}{x}_{j}\right)+\sum\:{b}_{ii}{{x}_{i}}^{2}+\:\sum\:{b}_{ij}{x}_{i}{x}_{j}$$

Where Y represents the value of the response variable (UTS, YS, EL%, HV), Xi represents the independent variables (Material type (M), Pin profile (P), cooling rate (C)) that are known for each experiment. The parameters b_o_, b_i_, and b_ij_ are the regression parameters. For all three parameters, the selected polynomial equation could be expressed as: -

2 $$\:Y={b}_{0}+{b}_{1}R+{b}_{2}T+{b}_{3}C+{b}_{11}{R}^{2}+{b}_{22}{T}^{2}+{b}_{33}{C}^{2}+{b}_{12}RT+{b}_{13}RC+{b}_{23}TC+\text{\pounds\:}$$

where b_1_, b_2_ and b_3_ are linear terms, b_12_, b_13_ and b_23_ are interactive terms, b_11_, b_22_, b_33_ are the quadratic terms of the polynomial and b_0_, b_1_, b_2_, b_3_, b_12_, b_13_, b_23_, b_11_, b_22_, b_33_ are the least square estimates of true polynomial, representing the response surface. The strength of the respective process parameters and their interactions is represented by these coefficients.

## Experimental work

### Material selection

Three different aluminum alloys (1050 AA, 6063 AA, and 2011 AA) were selected for experimentation under various processing conditions. Samples measuring 200 × 200 × 8 mm were cut from the selected alloys, with each sample used to conduct four experiments. These alloys’ chemical composition and mechanical properties are presented in Tables 3 and 4.

**Table 3:** Chemical composition of selected Alloys^49,50^.

Alloy/Element	Si	Mg	Cu	Zn	Fe	Ti	Mn	Pb	Bi	Cr	Al
1050 AA	0.25	0.05	0.05	0.07	0.6	0.04	0.05	-	-	-	Rest
2011 AA	0.4	-	5.3	0.3	5	-	-	0.4	0.35	-	Rest
6063 AA	0.43	0.6	0.12	0.1	0.35	0.04	0.09	0.4	-	0.1	Rest

**Table 4:** Mechanical properties of the as-received alloys.

Run	conditions	UTS	YS	El%	HV
AR 1050 AA	As received alloy	70.6	32.79	30	29
AR 2011 AA	As received alloy	235	130	16	90
AR 6063 AA	As received alloy	89.6	43.3	28	44

**Fig. 2:**
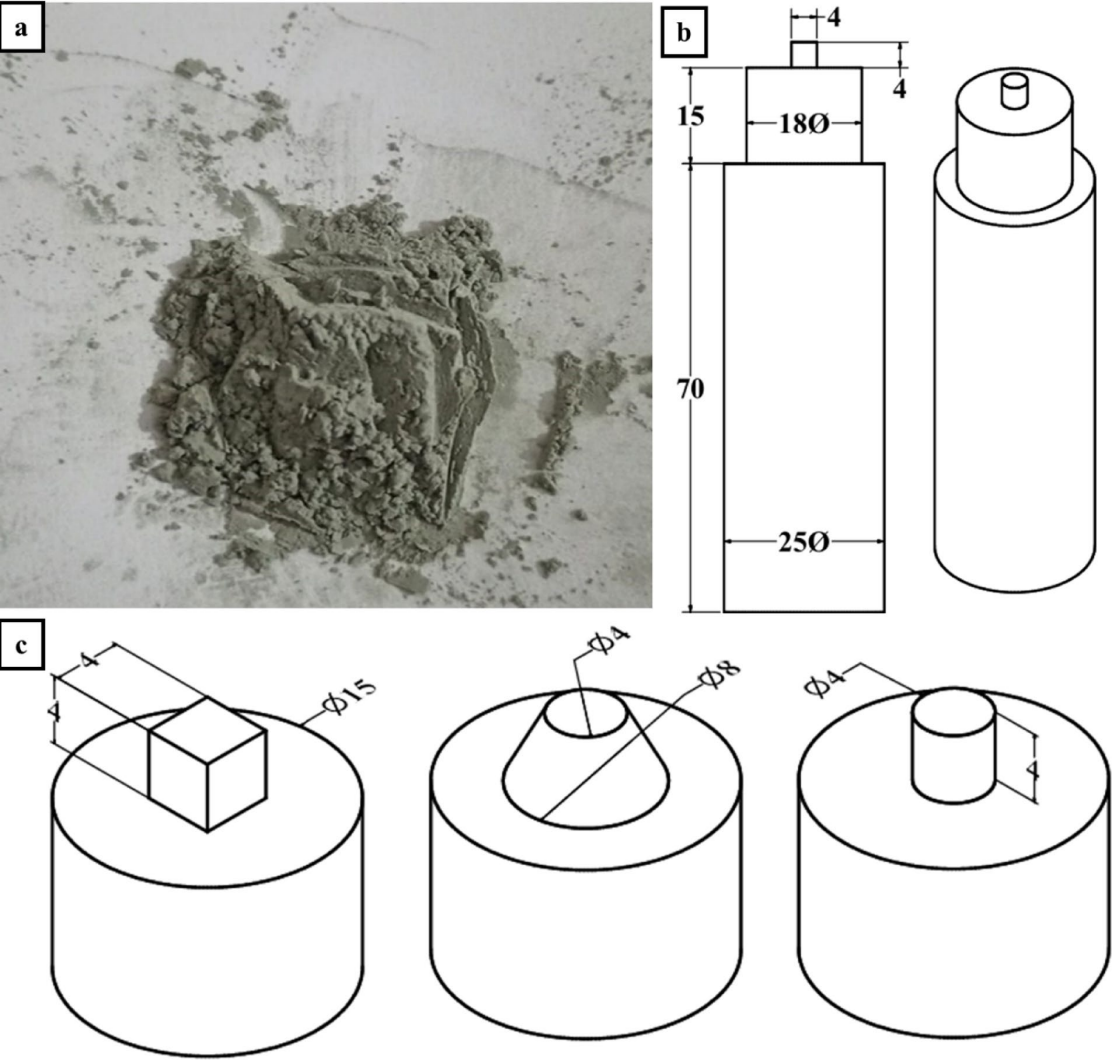
(**a**) Si_3_N_4_ particles, (**b**) Tool geometry, and (**c**) Pin profiles.

Silicon nitride (Si_3_N_4_) powders of average size equal to about 50 μm, as shown in Fig. 2a, were added as a reinforcement to aluminum alloys to enhance their mechanical properties and refine the grains of alloys.

### Friction stir processing

Friction stir processing in this work was carried out on a vertical milling machine. The milling cutter was replaced by a friction stir tool that was fixed in the chuck of the machine. The rotational speed of the tool during processing was selected to be 800 rpm, and the travel speed of the tool was determined to be 10 mm/min. Friction stir experiments were carried out with three different tool profiles. All tools, as shown in Fig. 2b, have an 18 mm shoulder diameter, coupled with a zero tilt angle, which was used to reduce the amount of material flow at the shoulder region^51,52^. Cylindrical, conical, and square pin profiles with flat surfaces, as shown in Fig. 2c, were selected as the main difference between stir tools.

The pin diameter is 4 mm for the cylindrical profile and 6 mm as the main diameter for the conical profile. The diameter of the cycle that touches the square from the nodes of the sides is 8 mm. The pin length of all profiles is 4 mm.

**Fig. 3:**
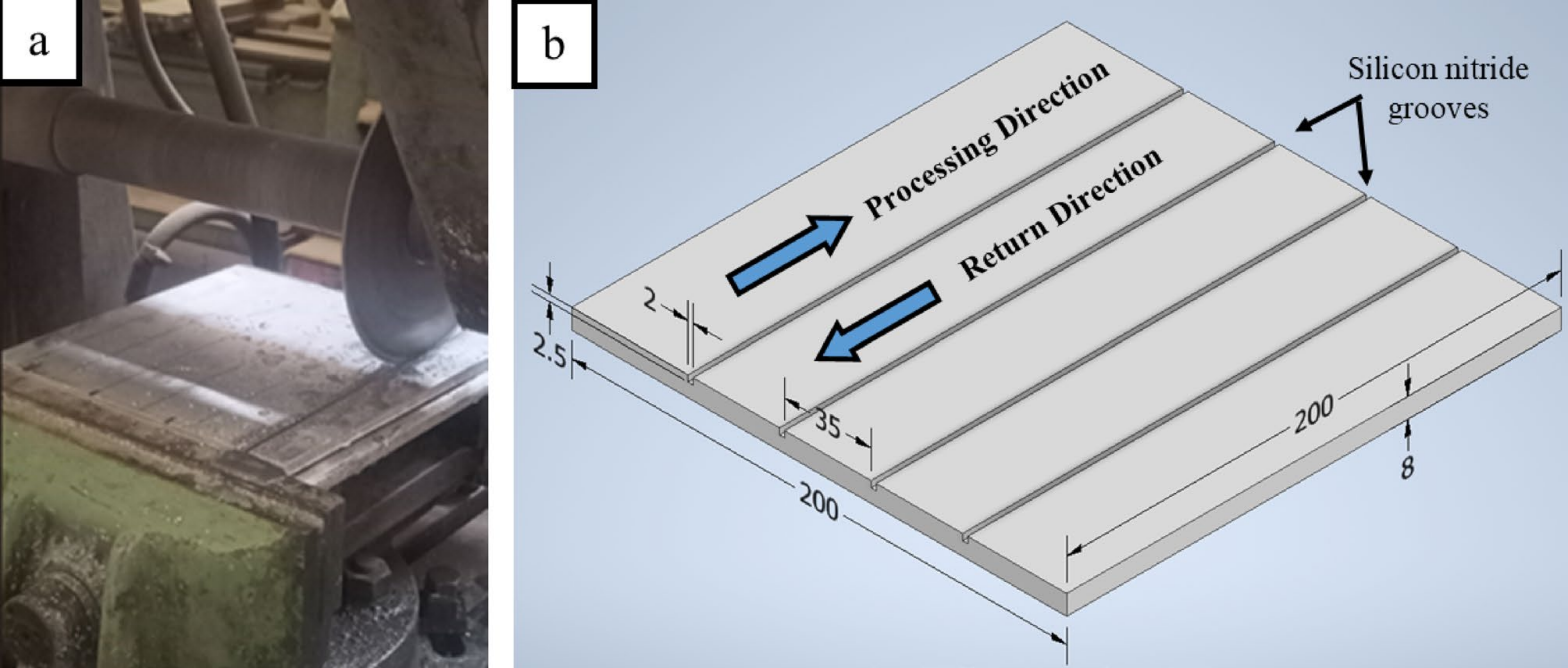
(**a**) Cutting powder groovers on the horizontal milling machine; and (**b**) Sample sketch.

Central grooves were made in different aluminum plates. These grooves were done on a peripheral milling machine as seen in Fig. 3a. Each groove has the same length of the FSP pass. These grooves are used for adding silicon carbide. Each groove has a 2 mm width and a 2.5 mm depth as shown in Fig. 3b. The average volume fraction of Si_3_N_4_ particles was about 10%. It was calculated by dividing the volume of the groove in the stir zone by the stir zone volume. A steel box covered by a copper plate filled with different cooling media (water and R-410a) was used in contact with the bottom surface of the Al sheet^53^. Samples from the base metal and FSP were prepared for microstructure evaluation. The specimens were ground using different grit papers with water as a cooling and ethylic alcohol as a cleaning agent between grinding steps. All specimens were polished using standard metallographic methods. The samples underwent etching with Keller’s etchant solution (190 ml H_2_O, 5 ml HNO_3_, 3 ml HCl, and 2 ml HF) to prepare for optical microscope (OM) examination.

**Fig. 4:**
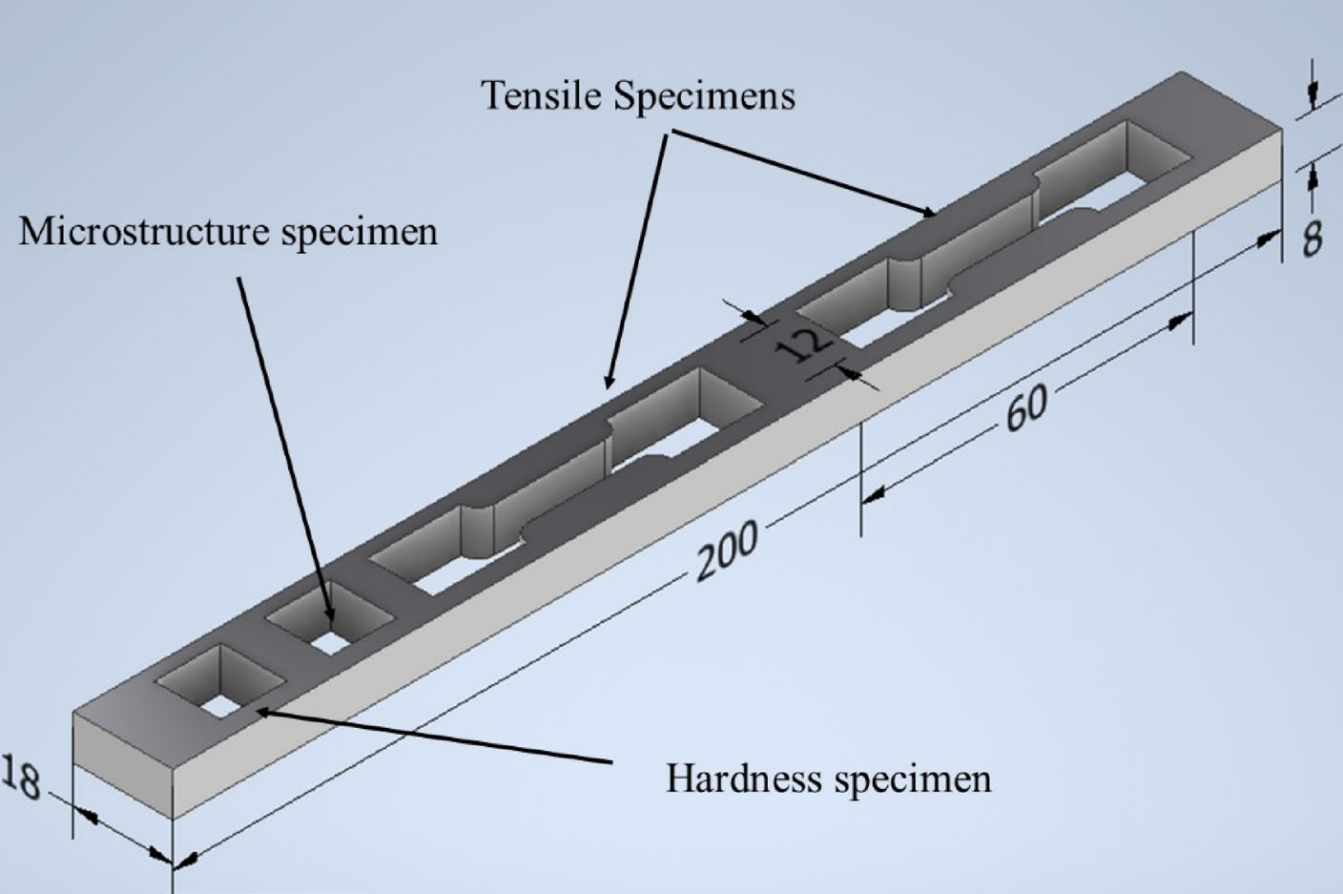
FSP pass schematic.

The tensile specimens, measuring 60 mm in length, 6 mm in width, and 2 mm in thickness, were cut according to ASTM E8/E8M, and parallel to the processing direction (FS pass) as shown in Fig. 4 with a wire cutting machine. The tensile test was performed using an INSTRON universal testing machine rated for 20 KN, with a crosshead speed set at 0.5 mm/min, at room temperature. Hardness was assessed using a Vickers hardness testing machine (Zwick-Roel-FV800). The samples were ground to create flat, parallel surfaces suitable for the testing stage. The hardness test adhered to standard procedures: a 300 g load was applied for 15 s through an indenter at a 136° included angle. The average result was calculated from 10 measured readings.

## Results

### Signal-to-noise ratio

The SN ratio is evaluated based on the quality of the intended responses. The objective function in this search is maximization of the responses (UTS, YS, El%, HV), so the larger the response, the better the SN ratio is to be evaluated. The formula used for calculating the SN ratio is given below.

$$\:SNratio=\:-10\:{\text{log}}_{10}\frac{1}{n}\sum_{i=1}^{n}\frac{1}{{{y}_{i}}^{2}}$$

The experimental values of the responses (UTS, YS, El%, HV) of the processed specimens under different process parameters (material type, pin profile, cooling medium) are converted into SN ratios as presented in Table 5. The response for the mean and SN ratio values for all levels is calculated and listed in Tables 6 and 7. A larger SN ratio corresponds to a better response. Based on mean and SN ratio values, the optimal level setting for UTS and Ys is M_2_ P_2_ C_1_, while the best setting for El% is M_1_ P_3_ C_1_. The best setting for HV is M_2_ P_3_ C_3_.

### Analysis of variance (ANOVA) in Taguchi

**Fig. 5:**
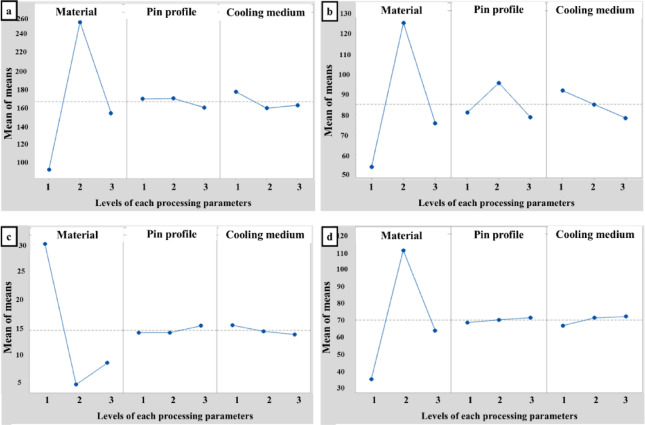
Main effects plot for mean of (**a**) UTS, (**b**) YS, (**c**) El%, and (**d**) HV.

**Fig. 6:**
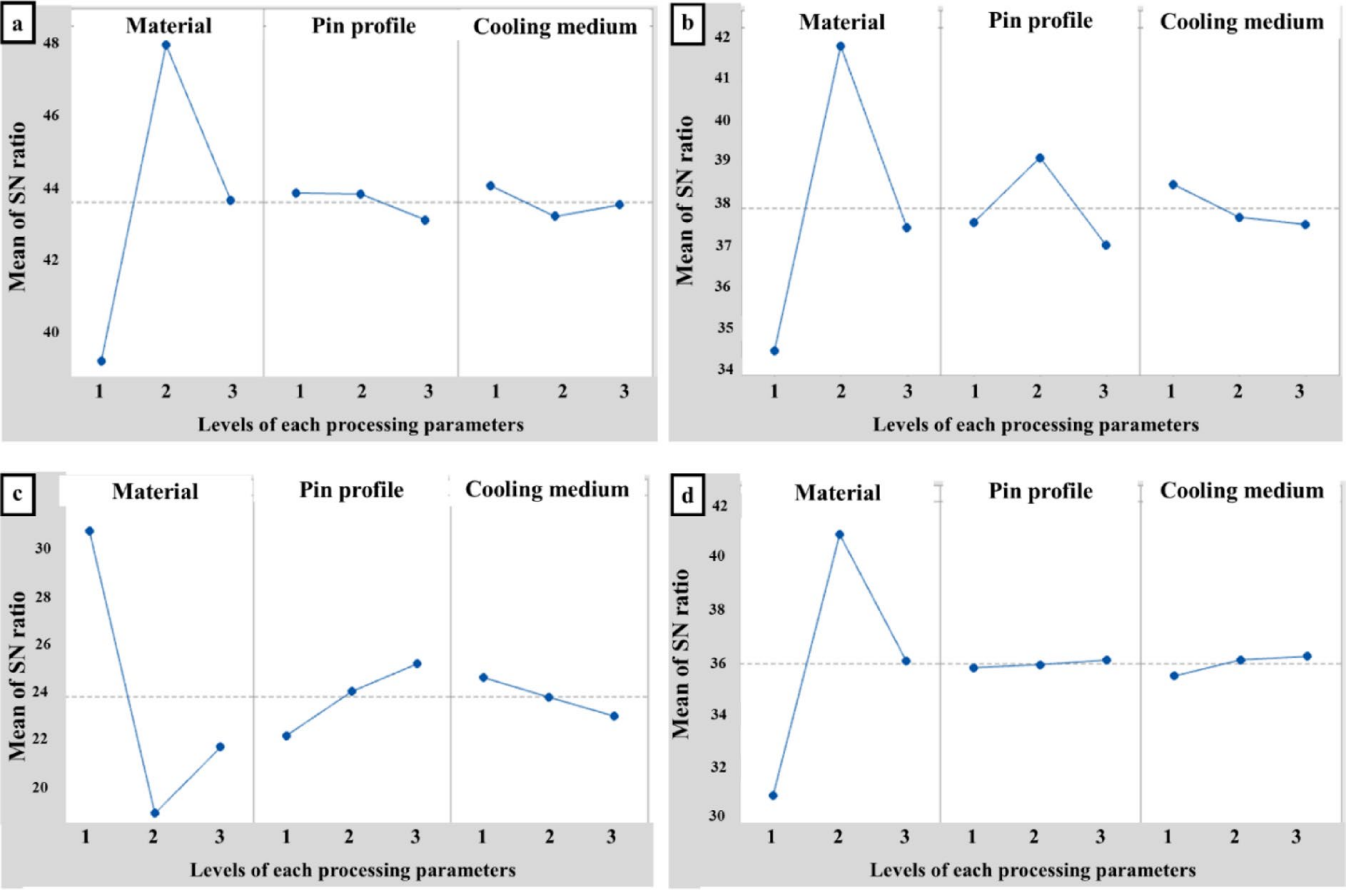
Main effects plot for SN ratio of (**a**) UTS, (**b**) YS, (**c**) El%, and (**d**) HV.

The purpose of ANOVA is to identify statistically significant factors. It provides a clear understanding of the extent to which the process parameter influences the response. The ANOVA tables for signal-to-noise ratio are calculated and presented in Tables 8, 9, 10 and 11. The main effects for the mean and SN ratio are shown in Figs. 5 and 6. The F-test is performed to assess the significance of the process parameter. A high F-value indicates that the factor significantly affects the process response. In this investigation, the type of material is a significant factor that plays a crucial role in determining the processed specimen’s UTS, YS, El%, and HV. On the other hand, the cooling medium and pin profile are significant only in the calculation of Ys and HV, respectively.

**Table 5:** Experimental layout – L_27_ orthogonal array, and SN ratio values.

Run No.	M Material	*P* Pin profile	C Cooling medium	UTS	Ys	El%	Hv	SNR1	SNR1	SNR1	SNR1
1	3	3	3	147	55	18	70	25.1055	34.8073	36.9020	43.3463
2	2	2	3	241	97	6	118	15.5630	39.7354	41.4376	47.6403
3	2	1	3	250	118	8	114	18.0618	41.4376	41.1381	47.9588
4	1	3	3	82	46	34	38	30.6296	33.2552	31.5957	38.2763
5	2	2	2	242	138	8	116	18.0618	42.7976	41.2892	47.6763
6	3	3	1	171	87	15	62	23.5218	38.7904	35.8478	44.6599
7	2	3	2	240	140	15	117	23.5218	42.9226	41.3637	47.6042
8	1	1	3	115	68	40	40	32.0412	36.6502	32.0412	41.2140
9	1	3	1	79	40	30	33	29.5424	32.0412	30.3703	37.9525
10	2	2	1	286	167	14	104	22.9226	44.4543	40.3407	49.1273
11	3	2	3	144	75	12	65	21.5836	37.5012	36.2583	43.1672
12	1	1	1	96	50	45	35	33.0643	33.9794	30.8814	39.6454
13	3	2	2	150	82	21	68	26.4444	38.2763	36.6502	43.5218
14	1	1	2	93	42	40	36	32.0412	32.4650	31.1261	39.3697
15	1	2	3	99	66	34	39	30.6296	36.3909	31.8213	39.9127
16	3	2	1	176	103	15	63	23.5218	40.2567	35.9868	44.9103
17	1	3	2	75	41	31	38	29.8272	32.2557	31.5957	37.5012
18	2	1	2	253	130	5	107	13.9794	42.2789	40.5877	48.0624
19	1	2	2	92	65	27	36	28.6273	36.2583	31.1261	39.2758
20	3	3	2	145	69	19	70	25.5751	36.7770	36.9020	43.2274
21	2	1	1	265	110	11	108	20.8279	40.8279	40.6685	48.4649
22	3	1	2	141	58	8	63	18.0618	35.2686	35.9868	42.9844
23	1	2	1	96	68	35	32	30.8814	36.6502	30.1030	39.6454
24	2	3	3	240	101	12	116	21.5836	40.0864	41.2892	47.6042
25	3	1	1	166	74	10	65	20.0000	37.3846	36.2583	44.4022
26	3	1	3	142	78	5	58	13.9794	37.8419	35.2686	43.0458
27	2	3	1	258	128	9	108	19.0849	42.1442	40.6685	48.2324

**Table 6:** Response table for signal to noise Ratios.

Level	UTS			YS			El%			HV		
	Material	pin profile	Cooling medium	Material	pin profile	Cooling medium	Material	pin profile	Cooling medium	Material	pin profile	Cooling medium
1	39.20	43.91	44.12	34.44	37.57	38.50	30.81	22.45	24.82	31.18	36.00	35.68
2	48.04	43.88	43.25	41.85	39.15	37.70	19.29	24.25	24.02	40.98	36.11	36.29
3	43.70	43.16	43.57	37.43	37.01	37.52	21.98	25.38	23.24	36.23	36.28	36.42
Delta	8.84	0.75	0.87	7.42	2.14	0.98	11.52	2.93	1.58	9.79	0.29	0.74
Rank	1	3	2	1	2	3	1	2	3	1	3	2

**Table 7:** Response table for Means.

Level	UTS			YS			El%			HV		
	Material	pin profile	Cooling medium	Material	pin profile	Cooling medium	Material	pin profile	Cooling medium	Material	pin profile	Cooling medium
1	91.89	169.00	177.00	54.00	80.89	91.89	35.111	19.111	20.444	36.33	69.56	67.78
2	252.78	169.56	159.00	125.44	95.67	85.00	9.778	19.111	19.333	112.00	71.22	72.33
3	153.56	159.67	162.22	75.67	78.56	78.22	13.667	20.333	18.778	64.89	72.44	73.11
Delta	160.89	9.89	18.00	71.44	17.11	13.67	25.333	1.222	1.667	75.67	2.89	5.33
Rank	1	3	2	1	2	3	1	3	2	1	3	2

**Table 8:** ANOVA table for SN ratio of UTS.

Source	DF	Adj SS	Adj MS	F- value	*P*-value	
Material	2	351.84	175.92	346.65	0.000	Significant
Pin profile	2	3.238	1.61	3.19	0.063	
Cooling	2	3.464	1.73	3.41	0.053	
Error	20	10.15	0.508			
Total	26	368.70				

**Table 9:** ANOVA table for SN ratio of YS.

Source	DF	Adj SS	Adj MS	F- value	*P*-value	
Material	2	431.554	215.77	1123.70	0.000	Significant
Pin profile	2	0.373	0.187	0.97	0.395	
Cooling	2	2.794	1.397	7.28	0.004	Significant
Error	20	3.840	0.192			
Total	26	438.562				

**Table 10:** ANOVA table for SN ratio of El%.

Source	DF	Adj SS	Adj MS	F- value	*P*-value	
Material	2	250.494	125.247	53.86	0.000	Significant
Pin profile	2	22.112	11.056	4.75	0.020	Significant
Cooling	2	4.913	2.456	1.06	0.366	
Error	20	46.508	2.325			
Total	26	324.027				

**Table 11:** ANOVA table for SN ratio of HV.

Source	DF	Adj SS	Adj MS	F- value	*P*-value	
Material	2	653.81	326.903	36.36	0.000	Significant
Pin profile	2	39.20	19.601	2.18	0.139	
Cooling	2	11.19	5.594	0.62	0.547	
Error	20	179.83	8.992			
Total	26	884.03				

Where DF - degrees of freedom, Seq SS – sequential sum of squares, Adj MS – adjusted mean square, SS0 – pure sum of squares, F – Fisher ratio.

### Regression model results

The experimental data presented in Table 12 were used to derive the regression model. From these regression models, the effects of friction stir process parameters against the independent variables (Material type (M), Pin profile (P), cooling rate (C)) on the responses (UTS, Ys, El%, and HV) of the friction stir process were evaluated. The independent parameters considered directly affect the generation of frictional heat and cause the plastic flow of the material. It was observed that when the combinations of parameters create either very low or very high frictional heat and material flow, a lower tensile strength is observed. The friction stir process results in the clustering of strengthening precipitates in the stir zone region.

**Table 12:** Mean values of output responses according to CCC design conditions.

Run	Conditions	UTS	St.Error	YS	St.Error	El	St.Error	Hv	St.Error
1	1050-Conical – water	92	2	65	2.0	27	1.8	36	2.9
2	2011- Conical – water	242	0.8	138	3.6	8	1.1	116	1.7
3	2011 - Conical – water	242	0.9	85.4	2.0	12	1.3	110	1.8
4	2011 - Square – water	240	8.8	140	3.6	15	1.1	117	2.3
5	2011 - Conical – water	237	6.1	106	1.7	7	0.9	111	1.1
6	2011 - Conical – water	242	0.8	112	2.2	10	0.9	109	1.0
7	2011 - Conical – water	237	6.0	122.4	1.5	7	0.3	113	2.2
8	2011 - Conical – air	286	6.5	167	2.6	14	0.5	104	1.5
9	6063 - Conical – water	150	4.3	82	1.2	21	0.6	68	1.0
10	2011 - Cylindrical – water	253	4.6	130	3.0	5	0.3	107	1.7
11	2011 - Conical – water	238	4.7	97	1.3	9	0.5	109	1.8
12	6063 - Square – air	171	3.9	88	2.4	15	1.7	62	1.0
13	1050 - Square – R-410a	82	2.3	46	2.6	34	0.8	38	0.6
14	6063 - Cylindrical – air	166	0.5	74	1.0	10	0.2	65	0.8
15	6063 - Cylindrical – R-410a	142	0.6	78	3.6	5	0.8	58	0.7
16	6063 - Square – R-410a	147	3.4	55	1.0	18	2.8	70	0.9
17	1050 - Cylindrical – air	96	4.4	50	1.7	45	1.7	35	0.8
18	1050 - Square – air	79	1	40	2.6	30	2.2	33	0.8
19	2011 - Conical – R-410a	241	5	97	1.7	6	0.4	118	1.0
20	1050 - Cylindrical – R-410a	115	2.78	68	2.6	40	1.2	40	0.7

### Effect of FSP parameters on the mathematical model

**Fig. 7:**
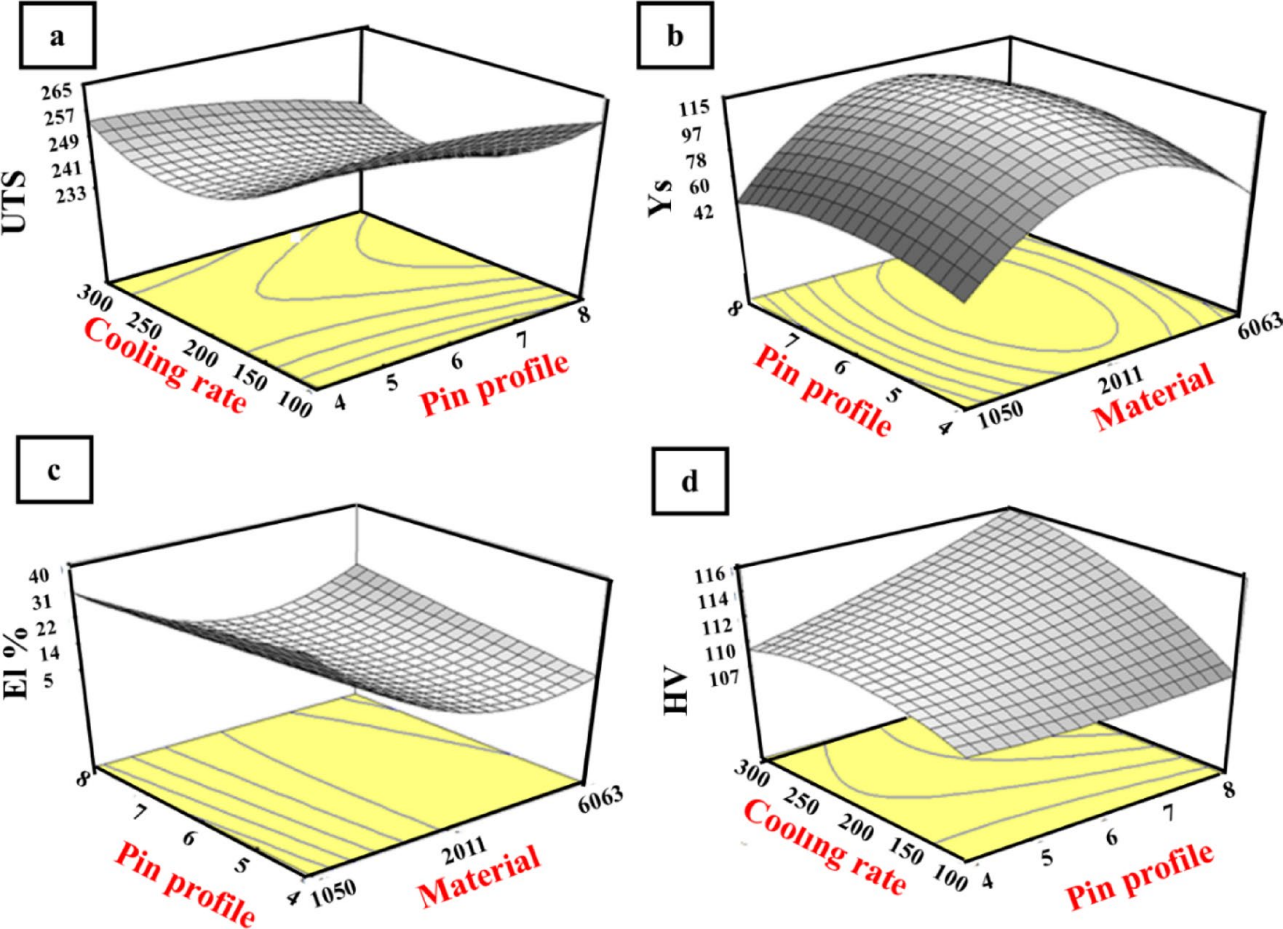
Effects of process parameters on the value of (**a**) UTS, (**b**) YS, (**c**) El%, (**d**) HV.

Figure 7 illustrates the impact of various process parameters (tool profile and cooling rates) on UTS, El, YS, and HV after the friction stir process. The maximum UTS, Ys values in 2011 AA are 286 and 167 MPa, respectively, with an increase of about 22% and 28% compared to as-received, where the maximum UTS and YS in 1050 AA were 115 and 68 MPa, with an increase of about 62% and 107% compared to as-received, but the maximum UTS and YS in 6063 AA are 171, 88 MPa, respectively, with an increase of about 90% and 50% compared to as-received. The increase of UTS and YS is related to the effect of FSP on the microstructural constituents, where FSP highly refines the microstructure.

**Fig. 8:**
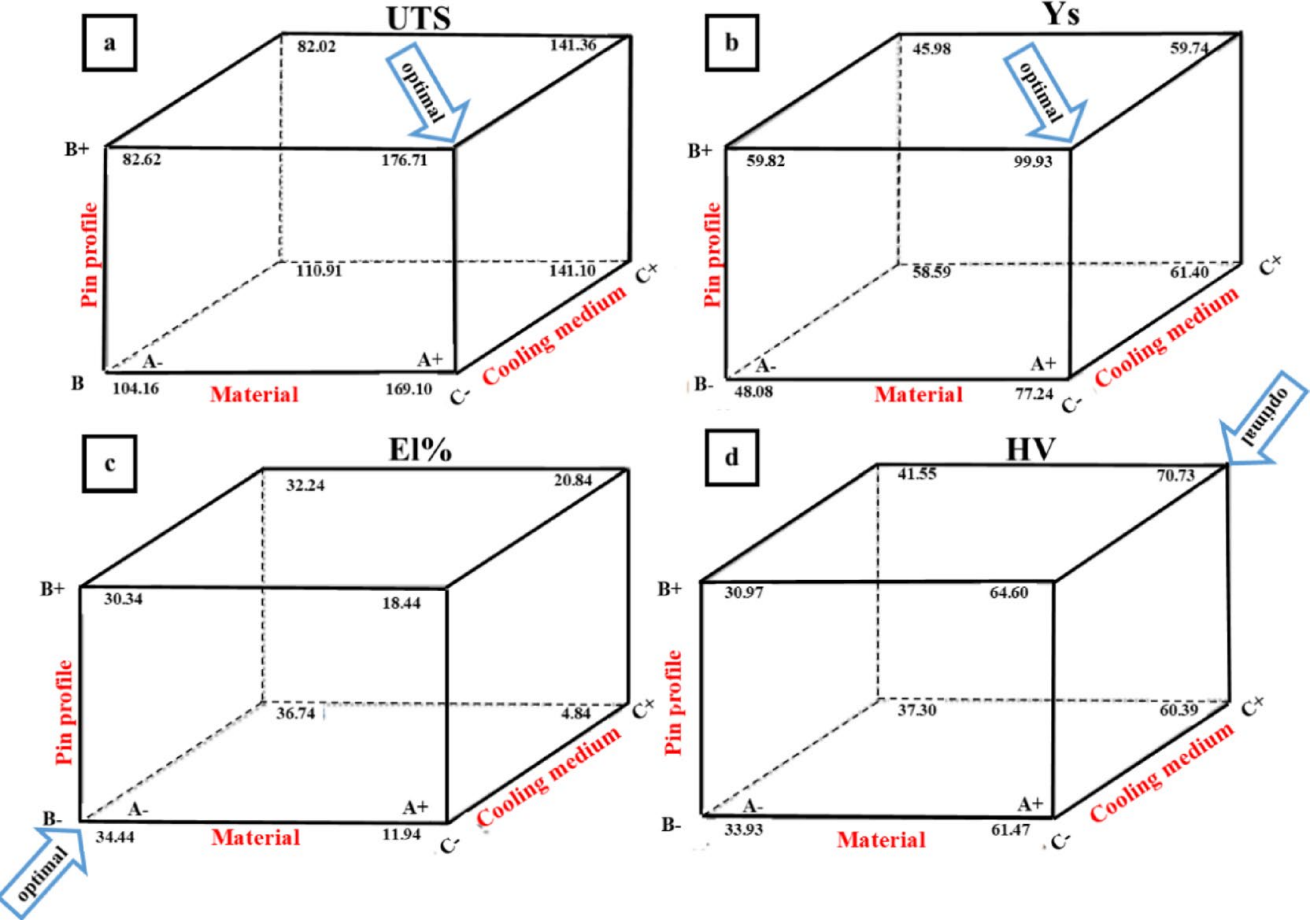
Cube graph for (**a**) UTS, (**b**) YS, (**c**) El%, and (**d**) HV.

The maximum hardness value is 118 HV, achieved by processing 2011 AA with a conical pin under indirect R-410a cooling media. This value represents an increase of about 24% compared to the as-received value, while the maximum hardness in 1050 AA is 40 HV, reflecting an increase of approximately 37% over the as-received value. In contrast, the highest hardness value in 6063 AA is 70 HV, with an increase of about 59% from the as-received value. The highest elongation percentage (El%) was observed by using a cylindrical pin on 1050 AA under indirect R-410a cooling media, reaching 40% and showing an increase of approximately 30% compared to its as-received value. All the experimental results (UTS, YS, Hv, and El%) were collected at the 3-D cubic, as shown in Fig. 8. Figure 8a reveals that UTS, ranging from 79 to 176 MPa, could be produced. For instance, a UTS value of 79 MPa can be obtained using 1050 AA processed by a square profile tool and under R-410a cooling media. Conversely, the highest UTS value of 175 MPa can be achieved with 6063 AA processed by a square profile tool and air-cooling medium.

### Microstructure and SEM analysis

During friction stir processing (FSP), the material in the processed region was heated locally to a high temperature, allowing for significant plastic deformation. This process causes effective mixing of the Si_3_N_4_ and redistribution of refined particles throughout the entire shear zone. Deformation primarily occurs at the tool pin interface, moving material from the front to the back. The shoulder of the tool stirs the material, mixing and relocating refined particles beneath it as it moves across the workpiece surface. The process results in dynamic recrystallization and enhanced refinement, yielding fine structures. These fine structures were prevented from coarsening due to rapid cooling by R-410a and water, which guarantees an effective cooling rate^54–56^. Additionally, a two-pass FSP with 100% overlap increases the number of small particles due to the low-temperature severe plastic deformation from the rotating tool. Consequently, very fine precipitates with a uniform distribution form within the stir zone, with the broken particles adopting a more rhombohedral shape^57^.

**Fig. 9:**
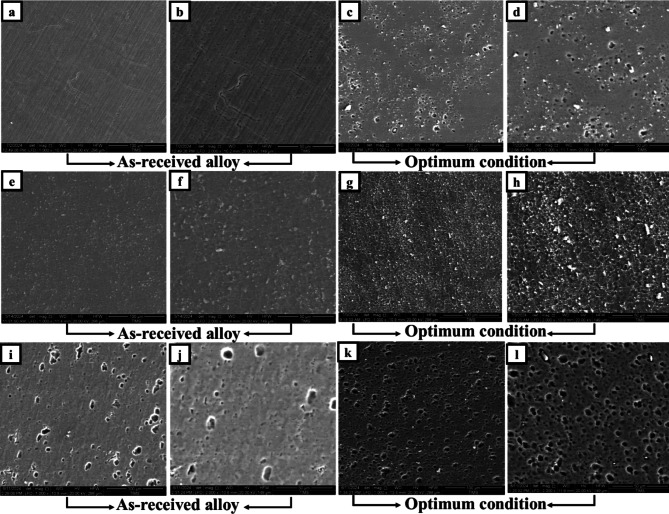
SEM photograph of grains after processing and addition of Si_3_N_4_ powders for: (**a-d**) for 1050 AA, (**e-h**) for 2011 AA, and (**i-l**) for 6063 AA.

Figure 9 presents SEM micrographs illustrating the distribution of Si_3_N_4_ particles within the produced aluminum alloys. It indicates that the grains in the FSP zone of the specimen were refined, leading to a significant plastic strain in the stirred zone, where severe plastic deformation was introduced into the workpiece by the rotating pin and shoulder. This process results in a redistribution of Si_3_N_4_ particles, which can be attributed to the heat generated by both the tool’s rotation speed and traverse speed, causing considerable dilution of the Si_3_N_4_ and aluminum. Achieving a homogeneous distribution of Si_3_N_4_ particles is crucial for enhancing the mechanical properties of the aluminum alloy composite (1050 AA, 2011 AA, 6063 AA) with Si_3_N_4_. The grain refinement mechanism in all alloys is dynamic recrystallization.

**Fig. 10:**
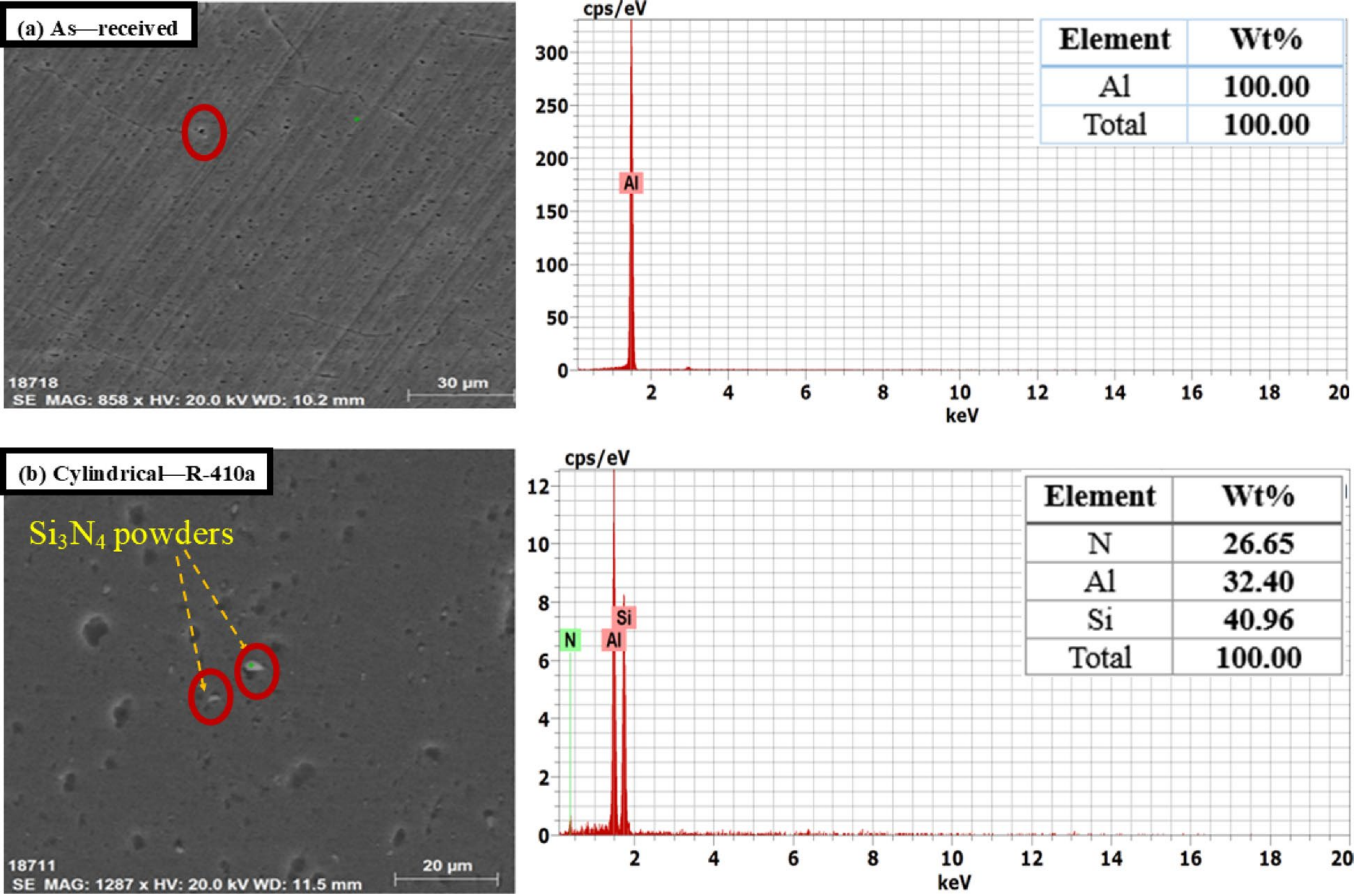
SEM and EDS of 1050 AA (**a**) As-received 1050 AA and (**b**) FSP with optimal condition.

**Fig. 11:**
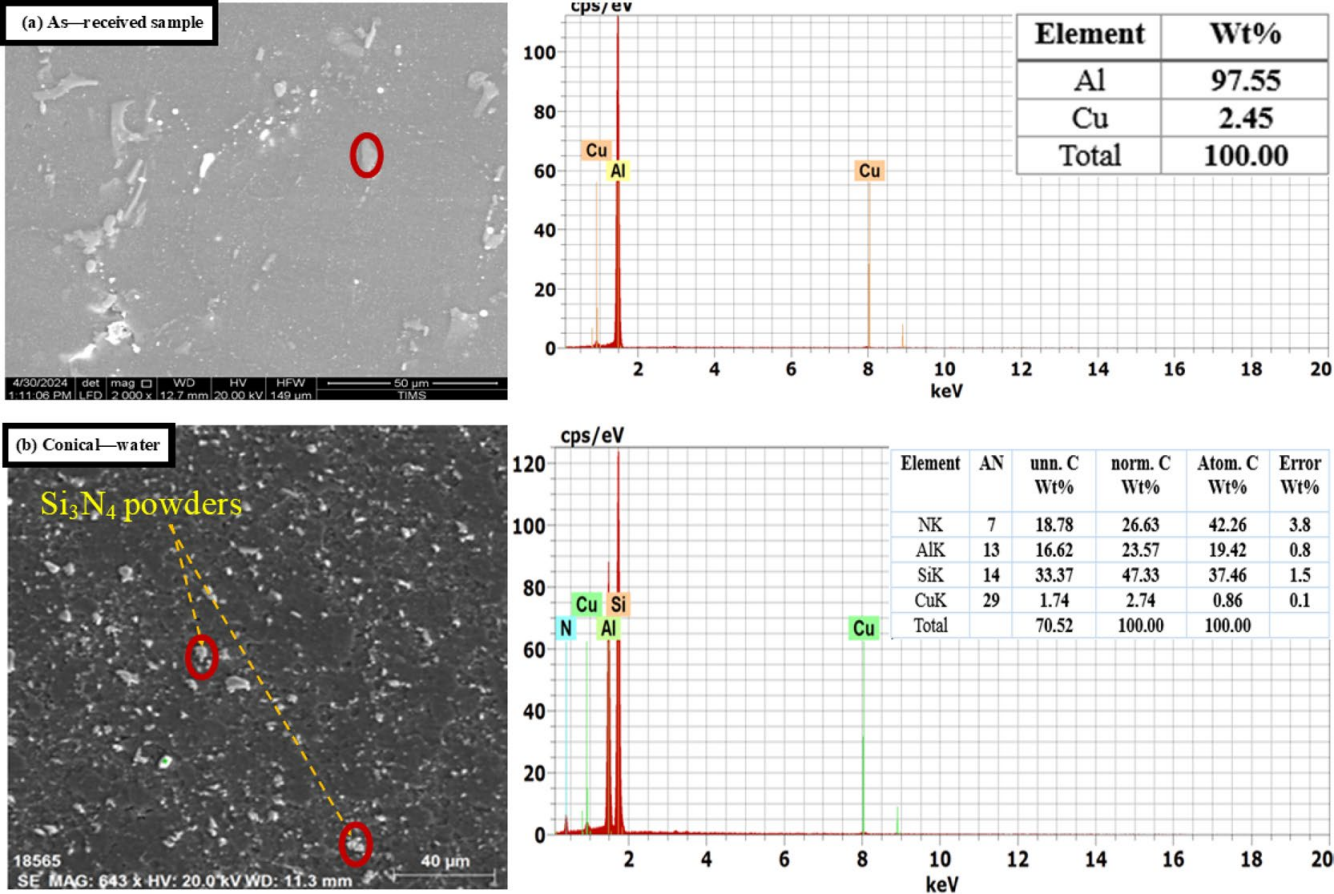
SEM and EDS of 2011 AA (**a**) As-received 2011 AA and (**b**) FSP with optimal conditions.

**Fig. 12:**
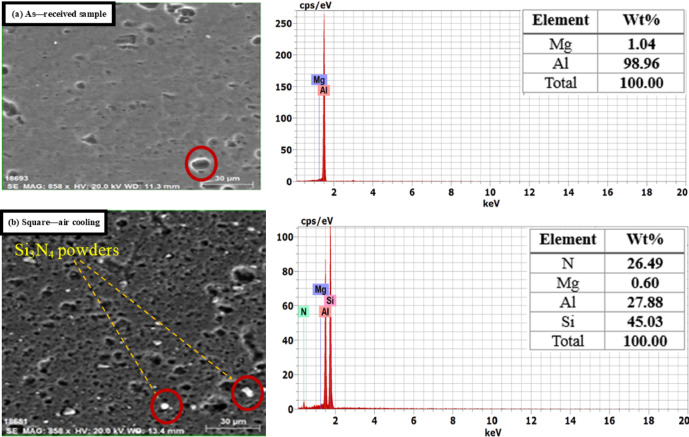
SEM and EDS of 6063 AA (**a**) As-received 6063 AA and (**b**) FSP with optimal conditions.

**Fig. 13:**
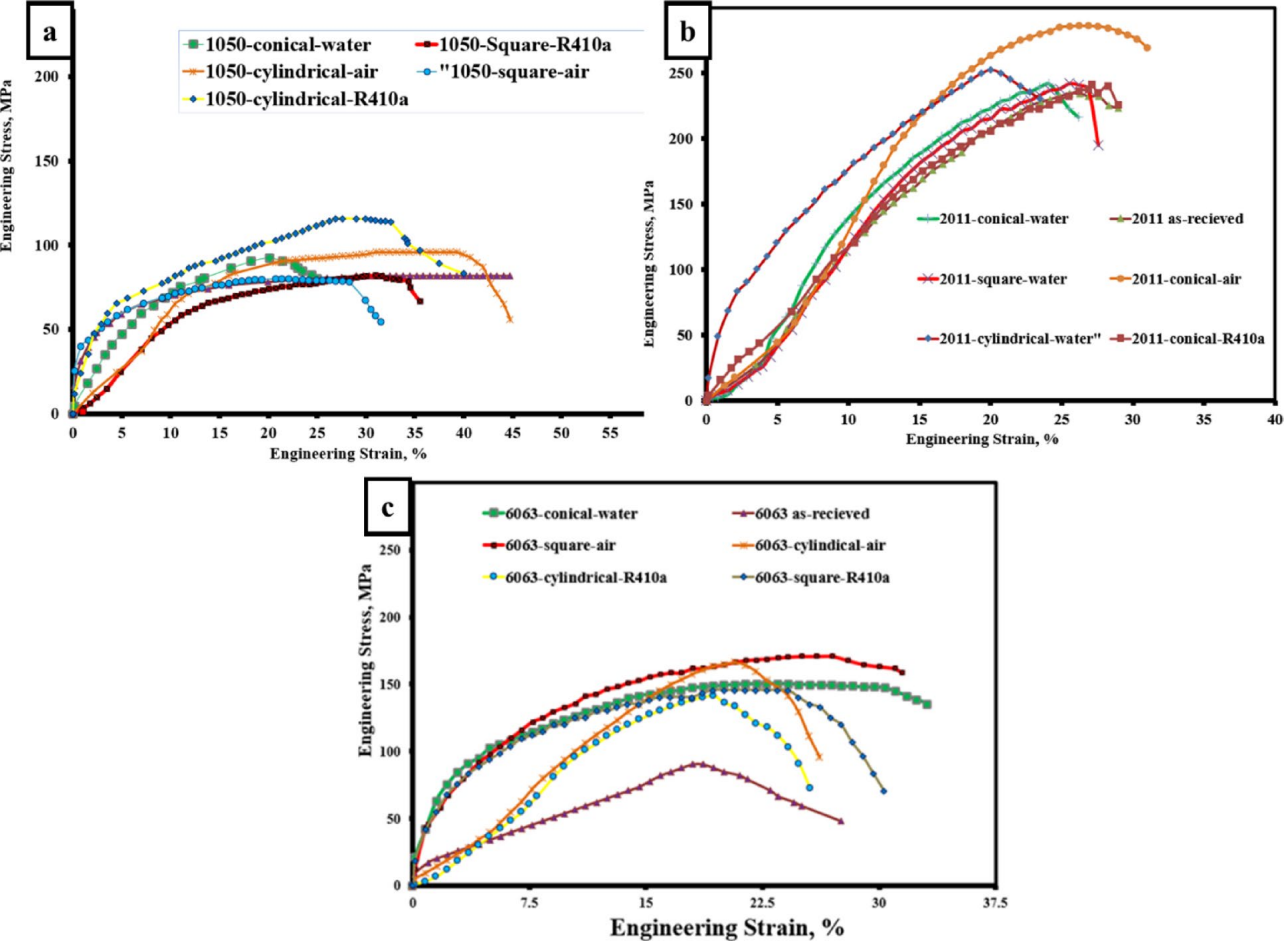
Stress-strain graphs for (**a**) 1050, (**b**) 2011, and (**c**) 6063 surface composites.

The EDS of specimens shown in Figs. 10, 11 and 12 confirmed the good powder distribution and revealed rich silicon powders (Si_3_N_4_). Good distribution of Si_3_N_4_ powders leads to an enhancement of the surface morphology of the intended alloys. The EDS pattern illustrated in the figures indicates that the resulting particles consist of elements such as Si, C, N, Mg, Cu, Mn, Fe, and S. The detection of these elements suggests that the debris contains components from both the tool and the backing plate (e.g., Fe, C). Additionally, the figures prove that elements like Cu, Mg, and Fe are present in the specimens, indicating that the worn particles and debris primarily originate from the tools or backing plate and may be associated with the dissociation of Si_3_N_4_. Nonetheless, tool wear debris is unavoidable during FSW/FSP, and it has been observed scattered within the layers of the FSP specimen^58,59^. As a result of the microstructure change, the mechanical properties of the fabricated surface composites improved. The stress-strain diagrams of some produced alloys are shown in Fig. 13.

### Checking for adequacy of the model

The ANOVA technique was used to assess the adequacy of the developed model. The results of the second-order response surface model were presented in the form of an analysis of variance. ANOVA results for ultimate tensile strength, yield strength, and percentage elongation are shown in Tables 13, 14, 15 and 16, respectively. If the calculated value of the F-ratio for the developed model is less than the standard F-ratio (from the F-table) value at a 95% confidence level, then the model is deemed adequate within that confidence level. The value of prob > F for the three developed models is less than 0.05 (95% confidence level), which indicates that the model is significant and that the lack of fit is also significant, as desired. All the coefficients were estimated and tested by applying an “F-test” using Design-Expert software to determine their significance at a 95% confidence level. After determining the significant coefficients, the final model was developed to predict the ultimate tensile strength (UTS) Eq. (3), yield strength (YS) Eq. (4), elongation percentage (El%) Eq. (5) and hardness value (HV) Eq. (6) of FSP of selected alloys as given below (coded equation).

3 $$\begin{gathered} {\mathbf{UTS}}\,=\,+\,{\text{242}}.{\text{95}}\,+\,{\text{31}}.0{\text{7 }}*{\text{ A}} - \,{\text{5}}.{\text{32 }}*{\text{ B}} - \,{\text{7}}.{\text{15 }}*{\text{ C}} - \,{\text{128}}.{\text{3}}0{\text{ }}*{\text{ }}{{\text{A}}^{\text{2}}} - {\text{2}}.{\text{95 }}*{\text{ }}{{\text{B}}^{\text{2}}}\,+\,{\text{14}}.{\text{3}}0{\text{ }}*{\text{ }}{{\text{C}}^{\text{2}}}\,+\,{\text{7}}.{\text{29 }}*{\text{ A }}*{\text{ B }} \hfill \\ \;\quad \quad \,\; - {\text{8}}.{\text{69 }}*{\text{ A }}*{\text{ C}} - \,{\text{1}}.{\text{84 }}*{\text{ B }}*{\text{ C}} \hfill \\ \end{gathered}$$

4 $$\begin{gathered} {\mathbf{YS}}\,=\,+\,{\text{114}}.{\text{3}}0\,+\,{\text{1}}0.{\text{73 }}*{\text{ A}}\,+\,{\text{2}}.{\text{52 }}*{\text{ B}} - \,{\text{7}}.{\text{42 }}*{\text{ C}} - \,{\text{47}}.{\text{55 }}*{\text{ }}{{\text{A}}^{\text{2}}}-{\text{14}}.00{\text{ }}*{\text{ }}{{\text{B}}^{\text{2}}}\,+\,{\text{11}}.{\text{1}}0{\text{ }}*{\text{ }}{{\text{C}}^{\text{2}}}\,+\,{\text{2}}.{\text{74 }}*{\text{ A }}*{\text{ B}} \hfill \\ \quad \quad - \,{\text{6}}.{\text{59 }}*{\text{ A }}*{\text{ C}} - \,{\text{6}}.0{\text{9 }}*{\text{ B }}*{\text{ C}} \hfill \\ \end{gathered}$$

5 $$\begin{gathered} {\mathbf{El}}\% =+{\text{ 9}}.{\text{12}}-{\text{1}}0.{\text{95 }}*{\text{ A}}\,+\,0.{\text{5}}0{\text{ }}*{\text{ B}} - \,{\text{1}}.{\text{3}}0{\text{ }}*{\text{ C}}\,+\,{\text{14}}.{\text{78}}*{\text{ }}{{\text{A}}^{\text{2}}}\,+\,0.{\text{53}}*{\text{ }}{{\text{B}}^{\text{2}}}\,+\,0.{\text{53}}*{\text{ }}{{\text{C}}^{\text{2}}}\,+\,{\text{5}}.{\text{13 }}*{\text{ A }}*{\text{ B}}\, \hfill \\ \quad \quad \;\;\;+\,0.{\text{13 }}*{\text{ A }}*{\text{ C}}\,+\,{\text{2}}.{\text{38 }}*{\text{ B }}*{\text{ C}} \hfill \\ \end{gathered}$$

6 $$\begin{gathered} {\mathbf{HV}}\,=\,+\,{\text{111}}.{\text{53}}\,+\,{\text{14}}.{\text{18 }}*{\text{ A}}\,+\,{\text{1}}.{\text{84 }}*{\text{ B}}\,+\,{\text{2}}.{\text{37 }}*{\text{ C}} - \,{\text{6}}0.{\text{12 }}*{\text{ }}{{\text{A}}^{\text{2}}}\,+\,0.{\text{44 }}*{\text{ }}{{\text{B}}^{\text{2}}}-{\text{1}}.{\text{73 }}*{\text{ }}{{\text{C}}^{\text{2}}}\,+\,{\text{1}}.{\text{52 }}*{\text{ A }}*{\text{ B }} \hfill \\ \quad \quad \; - {\text{1}}.{\text{11 }}*{\text{ A }}*{\text{ C}}\,+\,{\text{1}}.{\text{8}}0{\text{ }}*{\text{ B }}*{\text{ C}} \hfill \\ \end{gathered}$$

Where A denotes material type, B pin profile, and C cooling rate. From the above empirical equations, it is noted that the dominant parameter is material type.

**Table 13:** ANOVA table for UTS (Response surface quadratic Model).

Source	Sum of Squares	DF	Mean – Square	F-Value	Prob > F	
Model	85883.42	9	9542.603	90.52215	< 0.0001	Significant
Residual	1054.173	10	105.4173			
Lack of Fit	992.2049	5	198.441	16.01148	0.0043	Significant
Pure Error	61.96833	5	12.39367			
Cor Total	86937.6	19				

**Table 14:** ANOVA table for YS (Response surface quadratic Model).

Source	Sum of Squares	DF	Mean - Square	F-Value	Prob > F	
Model	15,265	9	1696.111	2.611494	0.0755	not significant
Residual	6494.794	10	649.4794			
Lack of Fit	4750.734	5	950.1469	2.723951	0.1478	not significant
Pure Error	1744.06	5	348.812			
Cor Total	21759.8	19				

**Table 15:** ANOVA table for el% (Response surface quadratic Model).

Source	Sum of Squares	DF	Mean - Square	F-Value	Prob > F	
Model	2665.171	9	296.1301	12.35744	0.0003	significant
Residual	239.6371	10	23.96371			
Lack of Fit	219.3288	5	43.86575	10.79994	0.0103	significant
Pure Error	20.30833	5	4.061667			
Cor Total	2904.808	19				

**Table 16:** ANOVA table for HV (Response surface quadratic Model).

Source	Sum of Squares	DF	Mean - Square	F-Value	Prob > F	
Model	20707.15	9	2300.794	136.6916	< 0.0001	significant
Residual	168.32	10	16.832			
Lack of Fit	134.4391	5	26.88782	10.967987	0.0083	significant
Pure Error	33.88093	5	6.776187			
Cor Total	20875.47	19				

### Residual analysis

**Fig. 14:**
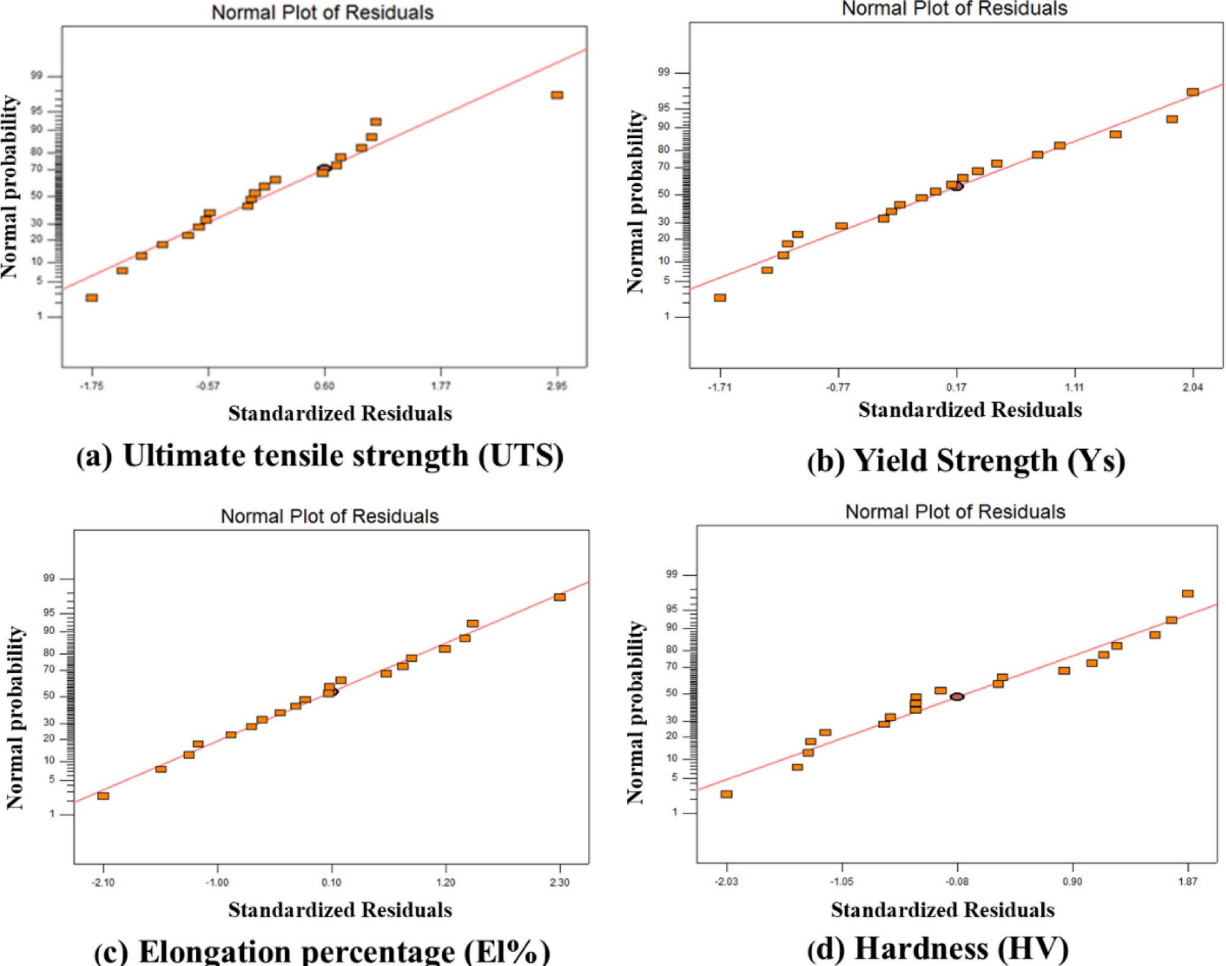
Normal plots of residuals for (**a**) UTS, (**b**) YS, (**c**) El%, and (**d**) HV.

A residual plot was created by plotting the residuals on the vertical axis and the independent variable on the horizontal axis. The quadratic regression could be considered appropriate for the obtained data if the points in a residual plot are randomly dispersed around the horizontal axis. On the other hand, if the points in the residual plot appear to be randomly scattered around zero, the vertical width of the scatter does not seem to increase or decrease across the fitted values, suggesting that the variance in the error term is constant. The normal percentage probability versus residual plots for yield strength, ultimate tensile strength, percentage elongation, and hardness values are shown in Fig. 14, which indicates that the residuals fall on a straight line, meaning that the errors are normally distributed. All the coefficients were calculated and verified by applying the ‘F-test’ using a trial version of Design-Expert software for their significance at a 95% confidence level.

### Conformity experimental tests for the developed models

The validity of the resultant regression equations was verified through experimental work. A conformity test was used to validate the process parameters’ level and check the reliability of the predicted and experimental values. Three test runs were conducted using the same experimental setup of process parameters, but with different values of those parameters. The error percentage was determined for the differences between the experimental and predicted outcomes, Eq. (7).

7 $$\% {\text{ Error }}={\text{ }}\left( {\left( {{\text{Av}} - {\text{ Pv}}} \right)/{\text{Pv}}} \right)/{\text{ 1}}00$$

where, Av = The actual value, Pv = The predicted value.

Table 17 presents a comparison between the experimental data, the predicted values and % Error depending on the input parameters obtained from Eq. 1 to 3 using quadratic RSMs. So, the quadratic models are very useful for predicting the ultimate strength, Yield strength, hardness, and elongation percentage.

## Conclusion

In this study, the Al/Si_3_N_4_ surface composites were produced by FSP using different tool geometries, cylindrical, square, and conical pin profiles, under different cooling mediums (air, water, R-410a). The Taguchi method and response surface methodology (RSM) were used to develop and analyze the ultimate tensile strength (UTS), percent elongation (El%), and proof strength (PS) of the produced aluminum surface composites (1050/Si_3_N_4,_ 2011/Si_3_N_4,_ and 6063/Si_3_N_4_) model. Si_3_N_4_ particles were added to the surface of these alloys. The Taguchi technique was employed to define the optimum levels of processing parameters (Material type (M), Pin profile (P), Cooling rate (C)), while the central composite design (CCD) in RSM, consisting of three variables, was adopted to develop the mathematical model for predicting the responses (UTS, Ys, El%, and HV) in the experimental study. An analysis of variance (ANOVA) was conducted to evaluate the model. The following points can be concluded:

- The FSP resulted in a significant breakup of coarse particles and primary aluminum dendrites, creating a homogeneous distribution of Si_3_N_4_ particles in the aluminum matrix. Good distribution of Si_3_N_4_ powders leads to an enhancement of the surface morphology of the intended alloys. These microstructural modifications significantly improved the tensile properties of the produced composites.
- The addition of Si_3_N_4_ powders to the surface of the base alloys plays a huge role in enhancing the hardness of the fabricated composites.
- The rapid cooling during FSP prevents grains from coarsening, leading to enhancement of the tensile properties, in particular ductility.
- The optimum level of settings was found using the Taguchi method. The optimal level setting for UTS and Ys is 2011 AA, conical pin profile, air cooling, while the best setting for El% is 1050 AA, square pin profile, air cooling. The best setting for HV is 2011 AA, square pin profile, and R-410a cooling.
- Regression models were developed based on the experimental values of yield strength, ultimate tensile strength, and elongation percentage from the friction stir process of different Al alloys. These models can predict the responses with a 95% confidence level.
- The regression equations detail the effects of operating parameters on yield strength, ultimate tensile strength, hardness, and elongation percentage of the friction stir process. The type of material is the most significant parameter affecting mechanical properties, while the type of cooling medium is the second most important parameter. The cooling media assist in reducing grain growth during FSP.
- The experimental and predicted values for UTS, Ys, El%, and HV align closely and fall within acceptable limits.
- The optimum UTS value predicted by the response surface model uses a 2011 AA, a conical pin profile, and air cooling during FSP. Conversely, the optimum Hv value from the response surface model uses a 2011 AA, a conical pin profile, and R-410 as cooling media during FSP.
- A maximum ultimate tensile strength of 286 MPa, yield strength of 167 MPa, elongation of 40%, and hardness of 118 HV were achieved.
- The observed values of mechanical properties showcase the promising prospects of Si_3_N_4_-reinforced aluminum composites for different industrial applications. The successful integration of Si_3_N_4_ reinforcement through Friction Stir Processing (FSP) demonstrates its potential to transform aluminum composite manufacturing, yielding superior mechanical properties that surpass those of conventional materials. This breakthrough opens up new possibilities for creating high-performance aluminum composites that are more durable and reliable, meeting the growing demand from various industries for advanced materials with exceptional characteristics.

**Table 17:** Actual, predicted and error values for response variables for UTS, YS and el%, and HV.

Standard Order	UTS			YS			EL%			HV		
	Actual Value	Predicted Value	% of Errors	Actual Value	Predicted Value	% of Errors	Actual Value	Predicted Value	% of Errors	Actual Value	Predicted Value	% of Errors
1	96	106.4	-10.4	49.6	49.1	0.4	47	46.2	0.7	34.44	32.8	1.6
2	166	166.5	-0.5	74	77.5	-3.5	10	10.2	-0.2	64.96	62.8	2.1
3	79	87.3	-8.3	40	45.4	-5.4	30	30.02	-0.02	33.34	31.4	1.9
4	170	175.0	-5.0	87.3	84.9	2.3	15	14.5	0.5	62.14	64.8	-2.6
5	114.5	106.1	8.3	68	73.4	-5.4	40	40.0	0	39.46	36.9	2.5
6	141.6	142.7	-1.1	78	75.6	2.3	5	4.5	0.5	57.72	60.2	-2.4
7	82	79.7	2.3	46	45.5	0.5	34	33.3	0.7	37.76	42.7	-4.9
8	146.4	143.9	2.5	55	58.5	-3.5	18	18.2	-0.2	69.93	69.4	0.6
9	91.8	83.5	8.2	64.9	54.8	10.0	27.5	28.8	-1.3	35.98	37.2	-1.3
10	150	145.7	4.3	81.5	79.1	2.4	21	21.3	-0.3	68.02	65.5	2.4
11	252.5	248.7	3.8	73.5	67.3	6.2	5	5.8	-0.8	106.63	110.5	-3.9
12	240	231.2	8.8	140	133.8	6.2	15	15.8	-0.8	118.48	113.4	5.0
13	286	261.5	24.5	167	160.8	6.2	14	14.8	-0.8	103.51	106.5	-3.0
14	241	252.9	-11.9	96.7	90.5	6.2	6	6.8	-0.8	117.26	113.0	4.2
15	241.3	242.9	-1.6	138	114.2	23.8	8	8.3	-0.3	115.63	111.5	4.0
16	237.2	242.9	-5.7	122.4	114.2	8.2	7.3	8.3	-1.0	112.71	111.5	1.1
17	233	242.9	-9.9	106	114.1	-8.1	6.5	8.3	-1.8	110.7	111.5	-0.8
18	237.6	242.9	-5.3	96.5	114.1	-17.6	9.5	8.3	1.1	108.86	111.5	-2.6
19	241.6	242.9	-1.3	111.7	114.1	-2.4	10	8.4	1.6	109.06	111.5	-2.4
20	242	242.9	-0.9	85.4	114.1	-28.7	12	8.4	3.6	109.9	111.5	-1.6

## Data Availability

Data supporting the conclusions are available upon reasonable request from the corresponding author.
